# Germination and seedling frost tolerance differ between the native and invasive range in common ragweed

**DOI:** 10.1007/s00442-013-2813-6

**Published:** 2013-11-07

**Authors:** Marion Carmen Leiblein-Wild, Rana Kaviani, Oliver Tackenberg

**Affiliations:** 1 Biodiversity and Climate Research Centre (BiK-F), Senckenberganlage 25, 60325 Frankfurt am Main, Germany; 2 Institute of Ecology, Evolution and Diversity, Goethe University Frankfurt, Max-von-Laue-Strasse 13, 60438 Frankfurt am Main, Germany; 3 Present Address: Michael Succow Foundation for the Protection of Nature, Ellernholzstrasse, 1/3, 17489 Greifswald, Germany

**Keywords:** Adaptation, *Ambrosia artemisiifolia* L., Intraspecific variation, Range expansion, Temperature

## Abstract

Germination characteristics and frost tolerance of seedlings are crucial parameters for establishment and invasion success of plants. The characterization of differences between populations in native and invasive ranges may improve our understanding of range expansion and adaptation. Here, we investigated germination characteristics of *Ambrosia artemisiifolia* L., a successful invader in Europe, under a temperature gradient between 5 and 25 °C. Besides rate and speed of germination we determined optimal, minimal and maximal temperature for germination of ten North American and 17 European populations that were sampled along major latitudinal and longitudinal gradients. We furthermore investigated the frost tolerance of seedlings. Germination rate was highest at 15 °C and germination speed was highest at 25 °C. Germination rate, germination speed, frost tolerance of seedlings, and the temperature niche width for germination were significantly higher and broader, respectively, for European populations. This was partly due to a higher seed mass of these populations. Germination traits lacked evidence for adaptation to climatic variables at the point of origin for both provenances. Instead, in the native range, seedling frost tolerance was positively correlated with the risk of frosts which supports the assumption of local adaptation. The increased frost tolerance of European populations may allow germination earlier in the year which may subsequently lead to higher biomass allocation—due to a longer growing period—and result in higher pollen and seed production. The increase in germination rates, germination speed and seedling frost tolerance might result in a higher fitness of the European populations which may facilitate further successful invasion and enhance the existing public health problems associated with this species.

**Electronic supplementary material:**

The online version of this article (doi:10.1007/s00442-013-2813-6) contains supplementary material, which is available to authorized users.

## Introduction

Invasive species are regarded as serious threats to global biodiversity (Sala et al. 2000). Their impact is expected to increase with climate change (Thuiller et al. 2006) including increases in species’ potential ranges (Kriticos et al. 2003). Thus, predicting the ecology and biogeography of invasive species is of increasing importance. The investigation of crucial life cycle traits and local adaptation is an important basis for understanding the process of range expansion, adaptation (Hierro et al. 2009) and community assembly (Gerhold et al. 2011). Specifically, comparative studies between populations from native and invasive ranges enable more precise conclusions on possible trait and niche shifts during the invasion process (Beckmann et al. 2011).

For many plant species, investigations which focus on traits such as growth, flowering phenology, and biomass have been done in either the invasive or native range (Gregor et al. 2013; Kollmann and Banuelos 2004; Montague et al. 2008; Weber and Schmid 1998). Conversely, so far only few comparative studies have considered traits related to germination and early establishment in both invasive and native ranges (Bossdorf et al. 2005).

Germination is of great importance, being the first step in a plant’s life cycle, and invasion success has been ascribed, amongst other factors, to high germination rates (Mandak 2003; Radford and Cousens 2000). Particularly for annual species, successful germination is crucial for the establishment of populations and range expansion. In this context, high germination rates and high germination speed are beneficial since successful establishment depends upon rapid exploitation of temporarily favorable conditions (Grime et al. 1981). Since the seedling stage is the most vulnerable time in the life cycle of a plant, the timing of germination is under strong selection: optimally, germination should occur only when the subsequent environmental conditions allow seedling establishment (Rathcke and Lacey 1985).

A trait highly associated with early establishment is frost tolerance of seedlings (Skálová et al. 2011) and many weedy plants are limited in their distribution by frost in temperate zones (Bruelheide and Heinemeyer 2002; Franklin 1995). Especially annual species with a long development period from germination to seed maturity are forced to germinate early in the year and may thus especially be endangered by spring frosts. Seedling frost tolerance may enable survival even after frost exposure. This is particularly relevant for an invader that is expanding its range northeastwards, as is the case in Europe for many plant species (Berger et al. 2007; Parmesan 2006), including also invasive species, e.g., Japanese knotweed (*Fallopia japonica*) or Cherry laurel (*Prunus laurocerasus*) (Berger et al. 2007; Dukes and Mooney 1999). However, so far frost tolerance of seedlings in native and invasive ranges has been investigated only rarely (Ebeling et al. 2008; Skálová et al. 2011).

During the process of invasion, genetic bottlenecks, founder effects and a loss of genetic variation commonly occur (Dlugosch and Parker 2008). Therefore, invasive ranges often only represent a subset of the existing variation of a species in a given trait. Consequently, ecological niches in the invaded range may be narrower due to the absence of some genotypes.

On the other hand, invasive species often exhibit a higher fitness in the invasive range compared to their performance in the native range. This finding is often interpreted as either evolutionary [e.g., the evolution of increased competitive ability (EICA) hypothesis (Blossey and Noetzold 1995)] or as an ecological response [e.g., enemy release hypothesis (Keane and Crawley 2002)] to altered abiotic factors or the lack of natural enemies in the invasive range. Higher fitness in the invasive range has been demonstrated for traits like germination, growth, resistance to herbivory etc. in a number of plant species (e.g., Erfmeier and Bruelheide 2005; Beckmann et al. 2011). Other mechanisms, such as selection by the introduction mode, could lead to altered traits in the invaded range. This may be relevant for *Ambrosia artemisiifolia*, which has been introduced as a seed contaminant and may be dispersed by humans over long distances. Species adapted to human activity may become especially successful invaders (Kowarik 2010). For a review on other hypotheses explaining the success of invasive species see, e.g., Kowarik (2010).

Intraspecific variation, i.e., the variation of characteristics or fitness-related measures between populations of a given species, has been demonstrated for various life history traits (Becker et al. 2006; Joshi et al. 2001), also in invasive ranges (Kollmann and Banuelos 2004; Montague et al. 2008). Such variation is often related to environmental conditions at the points of origin and interpreted as adaptation to these conditions. For germination, environmental factors like spring temperature conditions, altitude (Leger and Rice 2007; Skálová et al. 2011) or soil moisture (Rathcke and Lacey 1985) may be crucial.

In our study, we examined *A. artemisiifolia* (Asteraceae) as an example of a successful noxious plant invader in Europe. This annual plant species is native to North America where it grows in open grasslands or as competitive weed in crops. The first introduction of *A. artemisiifolia* seeds into Europe occurred in the nineteenth century as contaminants of wheat and other agricultural products from North America (Chauvel et al. 2006; Kowarik 2010). Today, the most important import vector is contaminated birdseed.

*A. artemisiifolia* grows successfully in crops or in disturbed areas, e.g., road verges or construction areas, in many European countries. The centre of the current distribution in Europe is in Southeastern Europe, the Po valley, and Southern France (Makra et al. 2004). A map of its current distribution is available at Cunze et al. (2013).

The wind-borne pollen of *A. artemisiifolia* may cause hay fever and dermatitis (Taramarcaz et al. 2005) resulting in associated costs of several million euros per year (Reinhardt et al. 2003). *A. artemisiifolia* only reproduces via seeds, which are viable for up to 39 years (Toole and Brown 1946). Compared to other annual species, *A. artemisiifolia* needs a long time to fulfil its life cycle: although one of the first annual species to germinate in the year (DiTommaso 2004), the first mature achenes are not expected until October (Kazinczi et al. 2008). Due to the requisite early germination, emerging seedlings are particularly endangered by frosts in spring. Mature *A. artemisiifolia* seeds are dormant and need stratification, normally supplied by winter conditions (Payne and Kleinschmidt 1961). It has been demonstrated that *A. artemisiifolia* populations from different geographic sites may differ in life history traits such as growth and flowering phenology (Hodgins and Rieseberg 2011; Leiblein-Wild and Tackenberg, under review), and that the species is genetically highly diverse (Genton et al. 2005). Therefore, it seems most likely that also a certain degree of variation in germination traits and frost tolerance might exist.

With our experiments we want to contribute to an understanding of the possible changes in germination patterns and frost resistance of invasive species. Especially, we want to enhance our understanding of *A. artemisiifolia*’s range expansion and local adaptation in germination parameters and frost tolerance to environmental conditions. Considering the aspects given above, we tested the following hypotheses with *A. artemisiifolia*:

1. Germination traits and frost tolerance differ between populations from the invasive (European) and native (North American) range.
2. Germination traits that affect the individual fitness, in particular germination rate and germination speed, are expected to be higher in the invasive range.
3. The germination niche of populations from the invaded range is expected to be narrower, e.g., due to genetic bottlenecks and missing genotypes.
4. Germination traits and frost tolerance are related to environmental variables of the respective point of origin in native and invasive ranges. We expect that the relationships between the considered traits and environmental parameters are stronger in the native range. Although common ragweed in North America is also dispersed by human activities and easily occurs in disturbed habitats, the species has had more time to occupy all possible niches and to adapt better to the respective local climate due to its residential time of many millennia.

## Materials and methods

### Germination experiment

We conducted a germination experiment with *A. artemisiifolia* populations from the invasive range in Europe and the native range in North America. In 2008 and 2009, seeds from 17 European and ten North American sites were collected. We sampled populations from 34.2°N (Georgia) to 44.1°N (Wisconsin) latitude and from −90.0°E to −76.4°E longitude in the native range, and from 44.0°N (France) to 50.1°N (Germany) latitude and from 4.3°E (France) to 19.7° (Hungary) longitude in the invasive range, to cover a broad geographical and environmental gradient. To avoid the use of temporary populations which might not exhibit any adaptation to local conditions, we only used populations from agricultural and ruderal sites that we are relatively sure are established and self-replicating. All points of origin of the populations and their environmental characteristics are listed in Table 1; maps of the sampled locations are given in the Electronic supplementary material (ESM; Fig. A1). Germination of *A. artemisiifolia* occurs only after seeds break dormancy, which should occur at ca. 4 °C (Willemsen 1975). Since Pickett and Baskin (1973) demonstrated higher germination rates with increasing length of stratification, we stored the seeds in a fridge at 4 °C for at least 5 months before starting the experiment.

**Table 1:** Geographic location, species traits and environmental characteristics of European and North American *Ambrosia artemisiifolia* populations ordered by latitude within the provenances

Pro	State	Region	ID	Latitude (°)	Longitude (°)	G-exp	F-exp	Seed mass (mg)	Temp. (°C)	Frost risk (days)	Elevation (m)
NA	Georgia	Rome	A39	34.222	−85.144	25	55	3.21	15.34	2.80	206
NA	Virginia	Blacksburg	A36	35.150	−81.520	25	52	3.09	15.13	3.99	218
NA	Arkansas	Blytheville	A8	35.961	−89.971		55	4.10	15.26	1.75	74
NA	Missouri	Kennett	A9	35.998	−89.994	25	45	3.21	15.26	1.75	74
NA	Virginia	Poquoson	A30	37.06	−76.400	25	62	2.68	14.98	0.95	3
NA	Virginia	Richmond	A28	37.713	−77.466	25		3.27	13.64	5.14	63
NA	Virginia	Covington	A27	37.811	−80.071		51	4.80	11.51	9.98	601
NA	Missouri	Ste. Genevieve	A10	37.918	−89.993	25	55	6.07	13.02	5.61	116
NA	West Virginia	Marmet	A24	38.286	−81.570	25	46	4.45	12.25	5.81	311
NA	Illinois	Divernon	A12	39.567	−89.716		58	6.33	11.68	6.55	194
NA	Indiana	Shirley	A21	39.852	−85.616	25	59	5.97	10.43	8.75	293
NA	Indiana	Remington	A19	40.761	−87.137	25		6.78	9.84	8.33	225
NA	Wisconsin	Beloit	A15	42.492	−88.987		63	5.13	8.29	9.84	264
NA	Wisconsin	Manitowoc	A16	44.052	−87.658	25	57	5.13	7.03	10.88	182
E	France	Valliguières	E12	44.013	4.587	50	40	6.13	12.73	1.00	187
E	France	Tharaux	E14	44.229	4.318	50		4.14	12.48	0.93	238
E	France	Granges-les-Beaumont	E9	45.049	4.984	50	37	6.67	11.95	5.64	174
E	France	Meyzieu	E6	45.771	4.988	50	38	5.13	11.49	3.22	195
E	Hungary	Ballószög	E27	46.883	19.557	25		4.68	10.78	3.74	114
E	France	Tart-l’Abbaye	E3	47.190	5.257	50	38	5.38	10.81	3.39	189
E	Hungary	Újhartyán	E25	47.212	19.389	25	45	5.27	10.84	3.07	113
E	France	Longeault	E2	47.217	5.529	50		4.48	10.63	3.22	221
E	Hungary	Budapest	E22	47.463	19.231	50		5.30	10.73	2.91	113
E	Hungary	Bag	E23	47.632	19.449	50	38	5.94	10.43	3.26	180
E	Hungary	Hatvan	E24	47.671	19.672	50		6.17	10.40	3.98	119
E	Germany	Karlsruhe	E17	49.044	8.393	25	39	4.00	10.44	3.63	107
E	Germany	St. Leon-Rot	E19	49.261	8.591	25		6.16	10.38	3.82	109
E	Germany	Mannheim	E20	49.534	8.466	25	39	10.28	10.12	4.35	94
E	Germany	Pfungstadt	E30	49.794	8.606	50	37	7.37	9.83	3.98	233
E	Czech Republic	Prague	E34	50.087	14.472	25	38	4.17	8.88	7.78	244
E	Germany	Höchst	E29	50.091	8.553	25		4.78	9.78	4.61	104
E	Germany	Sassleben	E32	51.754	13.973		39	6.91	9.20	7.12	94

*Pro* provenance, *NA* North America, *E* Europe, *ID* identification code of each population, *G-exp* number of seeds per petri dish used in the germination-tolerance experiment, *F-exp* number of seeds used in the frost-tolerance experiment, *Temp.* mean annual temperature (data source: worldclim.org), *Frost risk* mean number of days with frost between March and May [data sources: European Climate Assessment and Dataset (Haylock et al. 2008), National Climatic Data Center of the National Oceanic and Atmospheric Administration]

For each population and temperature regime we used five replicates. Each replicate consisted of 25 or 50 seeds from one population (depending on the available amount of seeds per population; for details see Table 1) which were placed into a petri dish (90 mm diameter; VWR International, Darmstadt, Germany) containing two filter papers wetted with 10 ml deionized water. Each petri dish was put into one of five growth chambers (RUMED, types 3401 and 3501; Rubarth Apparate, Laatzen, Germany) each with a different temperature regime (0/10, 5/15, 10/20, 15/25, and 20/30 °C), with a light/dark period of 12/12 h to simulate early spring conditions. In the following, each temperature regime is defined by its mean temperature, e.g., 0/10 °C is termed ‘5 °C’. The petri dishes were placed randomly inside the growth chambers and rotated regularly. If necessary, deionized water was added to ensure sufficient moisture and the petri dishes were then put back into the respective temperature regime.

Every 5 days the number of germinated seeds was counted for each petri dish and germinated seeds were removed. Seeds were classified as having germinated with the first visible protrusion of the radicle. To account for an observed low germination speed of North American populations and under low temperatures, the test period was set to 60 days.

### Germination parameters

In order to describe the relationship between temperature and germination rate, we primarily used a quadratic function that was fitted separately for each population (Eq. 1),

1 $${\text{germination rate}}=b_{ 0} + b_{1} *{\text{temperature}}+b_{2} *{\text{temperature}}^{ 2}$$

with *b*_0_, *b*_1_ and *b*_2_ being constants that were derived from curve fitting (IBM SPSS Statistics 20). An example of a quadratic function is shown in the ESM, Fig. A2. From these quadratic functions (which were only interpreted for positive germination rates), we directly calculated the following parameters that were used to characterize germination of each population in the subsequent analyses:

- Minimal temperature for germination (*T*_min_)
- Optimal temperature for germination (*T*_opt_)
- Maximal temperature for germination (*T*_max_)
- Niche width of the germination temperature (*T*_range_)
- Maximal germination rate (*G*_max_)

*T*_max_ and *T*_min_ were calculated via setting the quadratic function to zero, *T*_opt_ was calculated as the temperature value at the vertex of the respective quadratic function, and *G*_max_ was calculated as the peak germination rate at the vertex. *T*_range_ was calculated as *T*_max_−*T*_min_.

Furthermore, we used the time of 50 % germination (T50), i.e., the number of days when 50 % of seeds have germinated, to characterize the germination speed. T50 was calculated separately for each temperature and population: In order to get a measure of germination speed, which is independent from the total amount of seeds that finally germinate, the cumulative number of germinated seeds after 60 days of the respective population and temperature regime was defined as 100 % and T50 was calculated using linear interpolation (ESM, Fig. A3).

### Frost-tolerance experiment

To investigate the frost tolerance of *A. artemisiifolia* seedlings, we used seedlings from 11 European and 12 North American populations. If possible, we used the same populations as in the germination experiment. In the case of a lack of seed material, we used seed material of populations from similar latitudes (Table 1). For each population we germinated around 50 seeds under optimal (20/10 °C) temperature conditions with a 12/12 h dark/daylight cycle in a growth chamber (RUMED, type 3501; Rubarth Apparate).

After germination, seedlings were transplanted into seedling trays filled with propagating substrate (100 mg N l^−1^, 100 mg P_2_O_5_ l^−1^, 150 mg K_2_O l^−1^, 60 mg Mg l^−1^; C200; Stender, Schermbeck, Germany) and cultivated under the same temperature conditions until the appearance of the first pair of secondary leaves. Then the frost treatment was conducted. First, the temperature inside the climate chamber was set to 2 °C for 9 h to avoid temperature shock and to allow some acclimatization. Second, the temperature was decreased to −5 °C for 6 h to simulate night frosts in spring. Third, seedlings were cultivated at 2 °C again for 9 h, before returning to the optimal temperature conditions (20/10 °C). The next day, the state of each seedling (dead or damaged versus undamaged) was inspected visually and completely undamaged seedlings were classified as ‘frost tolerant’. In total we exposed around 50 seedlings per population (mean = 48, SD = 9.2; Table 1). From these data the proportion of frost-tolerant seedlings was determined for each population.

### Environmental variables at the point of origin

We considered environmental variables, such as temperature and the risk of frost exposure in spring, from the respective point of origin of the population. Monthly mean temperature values of current conditions (1950–2000) were derived from the WorldClim global climate database (www.worldclim.org/11.10.2010) with a spatial resolution of 1 km^2^. We used annual mean temperature in the analyses which, for our dataset, was highly correlated with the mean temperature in spring, when *A. artemisiifolia* germination takes place (Spearman *ρ* = 0.755, *P* < 0.001).

Data on frost occurrences during spring in Europe were derived from the European Climate Assessment and Dataset (Haylock et al. 2008) with a spatial resolution of 25 km^2^. The respective data for North America with a similar geographical resolution were derived from the National Climatic Data Center of the National Oceanic and Atmospheric Administration. We calculated the average number of days with a minimal temperature below 0 °C for each month between 1950 and 2012. The mean number of days per year with frost between March and May, i.e., the typical germination period of *A. artemisiifolia* (Kazinczi et al. 2008), was calculated for each point of origin and used as measure of the risk of spring frost exposure in the analyses. We also included latitude and longitude as explanatory variables. Latitude is highly associated with the length of day, which plays an important role in other stages of the ragweed life cycle, e.g., onset of flowering (Deen et al. 1998). Longitude may be seen as a rough measure of continentality, at least in Europe.

### Seed mass

For all populations used in the frost-tolerance experiment and the germination experiment, seed mass was determined. We took 50 randomly chosen seeds per population which were weighed simultaneously (CPA225D; Sartorius, Germany). Taking five replicates (consisting of 50 seeds) per population we calculated the mean mass for one individual seed.

### Statistical analysis

Differences in frost tolerance and germination traits (T50 at different temperature regimes, cumulative germination rates, *T*_min_, *T*_opt_, *T*_max_, and *G*_max_) between populations from the native and the invasive range were compared with a Mann–Whitney *U*-test.

As we found that mean seed mass differed between North American and European populations, we conducted an analysis of covariance (ANCOVA) to separate the effect of provenance and seed mass. Requirements for ANCOVA (normality of residuals, homoscedasticity) were given.

The relationship of germination traits and frost tolerance of *A. artemisiifolia* populations with the environmental variables and seed mass were calculated using Spearman rank correlations.

To compare the strength of correlations between native and invasive ranges, the analyses were conducted for each provenance (North America vs. Europe) separately.

All tests were computed with SPSS 20 (SPSS; Chicago, IL) or R (version 2.11.1; R Development Core Team 2011).

## Results

### Germination traits of European and North American populations

**Fig. 1:**
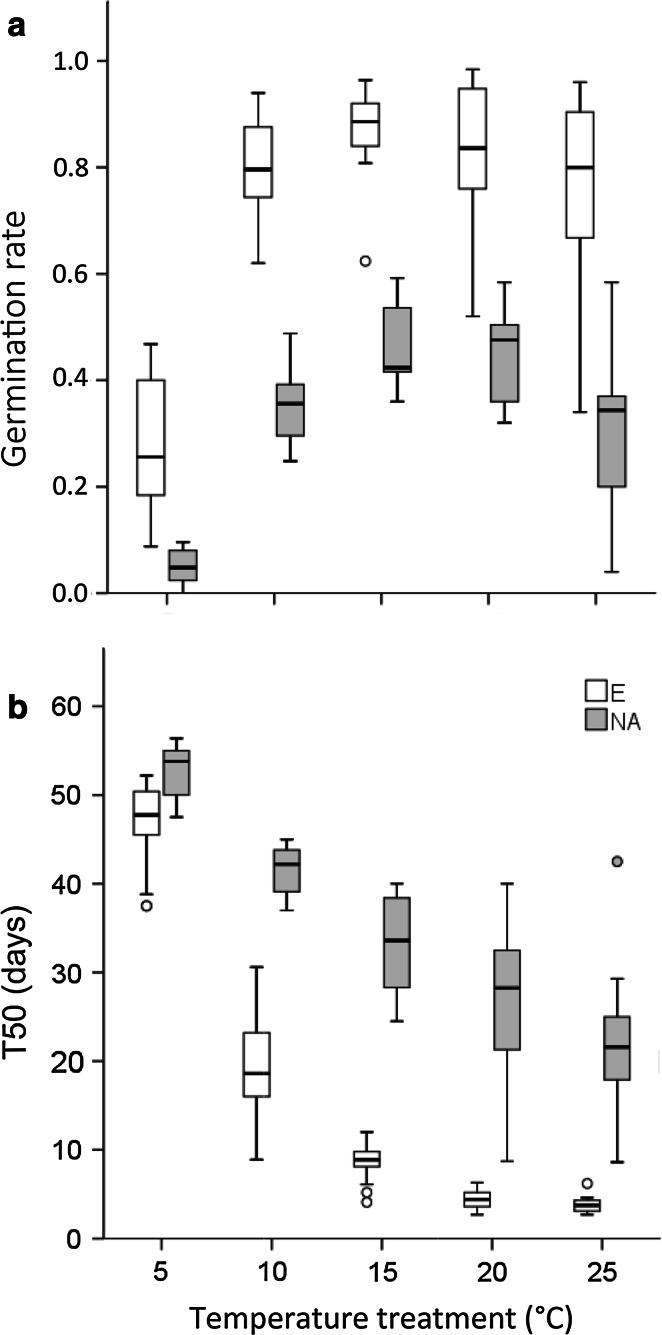
Box and whisker plots (*whiskers* with maximum 1.5 interquartile range) showing effects of temperature on germination of North American and European *Ambrosia artemisiifolia* populations. **a** Cumulative germination rates after 60 days and **b** number of days required to achieve 50 % of final germination (*T50*) of North American (*NA*; *n* = 10) and European (*E*; *n* = 17) populations

*A. artemisiifolia* L. germination occurred under all imposed temperature conditions between 5 and 25 °C, but T50 and germination rates differed considerably between the single temperature regimes: cumulative germination rates were highest under 15 °C conditions for populations from both provenances (Fig. 1a). Above and below this optimal temperature regime, germination rates decreased in populations from both ranges similarly (Fig. 1a). Under all five temperature regimes, cumulative germination of European populations was higher than in North American populations (Mann–Whitney *U*-test, *P* < 0.05; Fig. 1a).

T50 was lowest under 25 °C temperature conditions (mean of North American and European populations = 13.4 days) and increased continuously with decreasing temperature (Fig. 1b). T50 was significantly higher in North American populations than in European populations under each temperature (Table 2). Under low temperatures, seeds from the native range needed 5.9 days longer than seeds from the invasive range to achieve 50 % germination. Under optimal temperatures (15 °C) they needed 24.0 days longer, and under high temperatures (25 °C) the delay was 17.8 days.

**Table 2:** Differences in germination traits, frost-tolerance traits and seed mass of *A. artemisiifolia* from native (North American) and invasive (European) ranges

Parameter	Provenance		*U*	*P*
	Native range^a^	Invasive range^a^		
*T*_min_ (°C)	4.2 ± 0.63	2.0 ± 1.18	165	<0.001
*T*_max_ (°C)	30.6 ± 5.26	34.6 ± 4.55	45.5	0.046
*T*_opt_ (°C)	17.4 ± 2.71	18.3 ± 2.13	65	0.334
*T*_range_ (°C)	26.4 ± 5.19	32.5 ± 5.11	36	0.013
*G*_max_ (%)	48.9 ± 13.07	91.3 ± 11.60	3	<0.001
T50_5 (days)	52.6 ± 3.46	46.8 ± 4.94	128	0.004
T50_10 (days)	41.7 ± 2.84	19.2 ± 5.45	170	<0.001
T50_15 (days)	33.3 ± 5.44	9.3 ± 3.64	170	<0.001
T50_20 (days)	26.3 ± 9.28	5.2 ± 3.53	168	<0.001
T50_25 (days)	22.2 ± 9.24	4.5 ± 2.72	168	<0.001
Frost tolerance (%)	23.3 ± 7.8	37.0 ± 12.8	114.5	0.003
Seed mass G-exp (mg)	4.4 ± 1.50	5.7 ± 1.52	46	0.052
Seed mass F-exp (mg)	4.5 ± 1.27	6.1 ± 1.74	29	0.023

Seed mass is shown for the subset of populations used in the germination experiment (G-exp) and for the subset used in the frost-tolerance experiment (F-exp)

*T*_min_ minimal temperature for germination, *T*_max_ maximal temperature for germination, *T*_opt_ optimal temperature for germination, *T*_range_ niche width of the germination temperature, *G*_max_ maximal germination rate,* T50* time at which 50 % germination is achieved

^a^Mean values ± SD

The quadratic models explaining the relationship between temperature and cumulative germination were highly significant (*P* < 0.001) and displayed very high *R* ^2^-values (mean = 0.82 ± 0.08; ESM, Table A1) for each population. Thus the models seem appropriate to calculate the derived statistics *T*_min_, *T*_opt_, *T*_max_ and *G*_max_.

*T*_opt_ varied from 13.8 to 21.8 °C and did not differ significantly between native and invasive provenances (Table 2). *T*_max_ ranged from 23.6 to 40.3 °C and was significantly higher in populations from the invasive range (Table 2). *T*_min_ was on average 3.1 °C and significantly lower in populations from the invasive range (Table 2). Consequently, the temperature niche (*T*_range_) was significantly higher in populations from the invasive range than in native ones (Table 2). *G*_max_ averaged over all populations was 70.1 % and was significantly higher for populations from the invasive range (Table 2). Seeds used in the germination experiment tended to be heavier in the invasive range compared to the native range, although the difference was not quite statistically significant (Table 2).

### Frost tolerance of European and North American populations

Frost tolerance, i.e., the proportion of undamaged seedlings after frost treatment in the climate chamber, was higher in European (37.0 ± 12.8 %) than in North American populations (23.3 ± 7.8 %, Mann–Whitney *U*-test, *P* = 0.003; Table 2). Frost tolerance was less variable within the native *A. artemisiifolia* populations (12.9 to 36.8 %) compared to that of European populations, which varied from 7.9 to 56.8 %. The mass of seeds used in this experiment was significantly higher for the invasive than for the native range (Table 2).

### The influence of seed mass on germination traits and frost tolerance

**Fig. 2:**
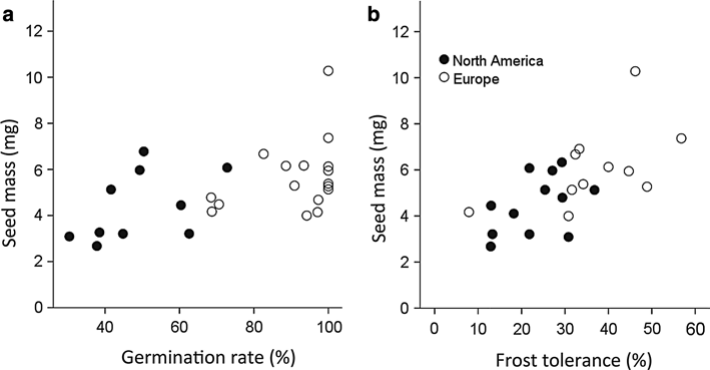
Relationship between **a** germination rate (*G*_max_) with seed mass (native range, Spearman *ρ* = 0.624, *P* = 0.054, *n* = 10; invasive range, *ρ* = 0.340, *P* = 0.181, *n* = 17) and **b** frost tolerance with seed mass (native range, *ρ* = 0.378, *P* = 0.225, *n* = 12; invasive range, *ρ* = 0.636, *P* = 0.035, *n* = 11) for *A. artemisiifolia* populations from native and invasive ranges

On average, seed mass was higher for populations from the invasive range (significantly higher for the subset of populations used in the frost-tolerance experiment and higher by trend for the subset of populations used in the germination experiment). To account for the possible influence of seed mass, data on germination traits and frost tolerance were analyzed using ANCOVA to separate the effect of provenance and seed mass. Besides provenance, seed mass also had a significant effect on *G*_max_, *T*_max_, *T*_range_, and frost tolerance of seedlings (Table 3). The positive correlation between seed mass and *G*_max_, as well as the correlation between seed mass and frost tolerance, are shown as exemplary (Fig. 2). However, usually more variance was explained by the provenance (Table 3). Seed mass had no influence on *T*_min_ and T50, which differed significantly between the native and invasive range (Table 3).

**Table 3:** Effect of provenance (North America versus Europe) and seed mass on germination traits and frost tolerance of seedlings tested using analysis of covariance

	*T*_min_			*T*_opt_		*T*_max_		*G*_max_		*T*_range_		T50_15		Frost tolerance		
	*df*	*F*	*P*	*F*	*P*	*F*	*P*	*F*	*P*	*F*	*P*	*F*	*P*	*df*	*F*	*P*
Provenance	1	26.97	<0.001	1.39	0.250	5.66	0.026	84.76	<0.001	10.78	0.003	194.67	<0.001	1	12.21	0.002
Seed mass	1	0.46	0.503	12.16	0.002	9.46	0.005	4.42	0.047	6.78	0.016	2.68	0.115	1	7.26	0.014
Residuals	23													19		

For abbreviations, see Table 2

### Correlations of germination traits and frost tolerance with geographical and climatic variables

For the native range, most germination traits were not correlated with any of the considered environmental parameters (Table 4). Only *G*_max_ was negatively correlated with longitude, indicating that populations from eastern, more oceanic provenances, exhibit lower germination rates. For the invasive range, we found no correlation of germination traits with geographical and climatic variables (Table 4).

**Table 4:** Correlation coefficients (Spearman *ρ*) of germination traits and frost tolerance of *A. artemisiifolia* populations with environmental parameters of the point of origin for the native and invasive range

Trait	Latitude	Longitude	Temperature	Frost risk	*n*
Native range (North America)					
*T*_min_	0.429	−0.288	−0.472	0.436	10
*T*_max_	0.539	−0.527	−0.479	0.479	10
*T*_opt_	0.539	−0.527	−0.479	0.479	10
*G*_max_	0.261	−0.758*	−0.164	0.212	10
T50_15	−0.309	0.188	0.358	−0.455	10
FT	0.322	0.084	−0.526†	0.726**	12
Invasive range (Europe)					
*T*_min_	0.301	0.124	−0.252	0.115	17
*T*_max_	−0.324	−0.151	0.318	−0.356	17
*T*_opt_	−0.227	−0.150	0.238	−0.352	17
*G*_max_	−0.340	−0.041	0.353	−0.417^‘^	17
T50_15	−0.339	−0.398	0.280	−0.086	17
FT	0.000	0.273	0.018	−0.364	11

*FT* Frost tolerance, *ρ* Spearman correlation coefficient, *n* number of populations; for other abbreviations, see Table 1

†* P* < 0.1, * *P* < 0.05, ** *P* < 0.01

**Fig. 3:**
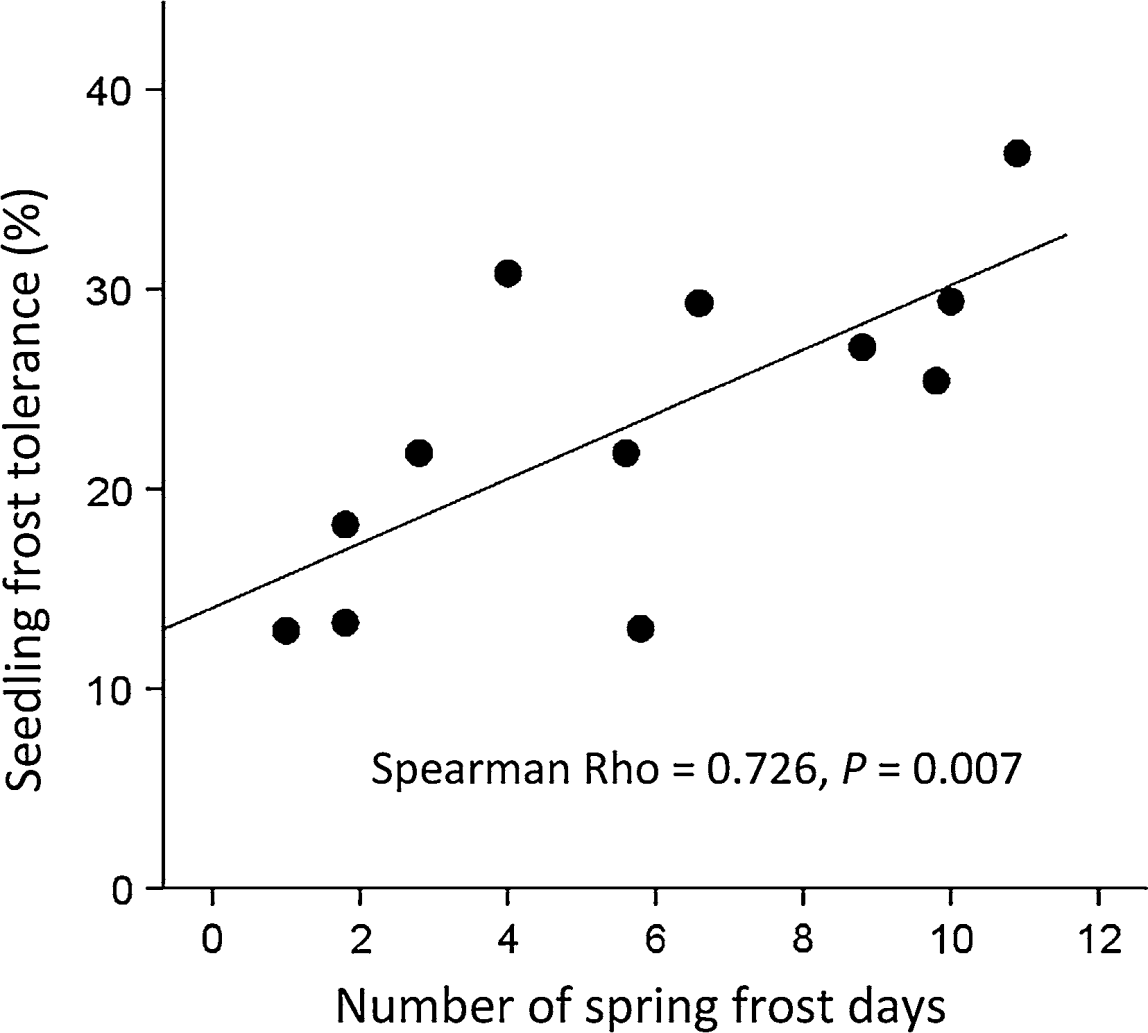
Frost tolerance of seedlings for North American *A. artemisiifolia* populations in relation to the risk of spring frosts at the respective point of origin, *n* = 12

Frost tolerance of seedlings was strongly positively correlated with the risk of frost occurrence in spring for the native range (Fig. 3; Table 4). For populations from the invasive range, no correlation of frost tolerance with environmental parameters was found.

## Discussion

### Temperature demands for germination

We investigated the germination of *A. artemisiifolia* along a temperature gradient from 5 to 25 °C and found that germination occurred under all temperature regimes. *T*_opt_ did not differ between European and North American populations and ranged from 13.8 to 21.8 °C with most populations having an optimum between 16 and 17 °C. These results are in accordance with observations from Brandes and Nitzsche (2007) who found an optimal germination temperature between 15 and 25 °C. While Forcella et al. (1997) observed 13 °C as a minimum temperature for *A. artemisiifolia* germination, we demonstrated a lower minimal temperature: in our experiment, seeds germinated at temperatures of 5 °C (lowest temperature regime). The calculated *T*_min_ values (mean = 3.1 °C) for germination are in accordance with Guillemin et al. (2013) who found a base temperature of 3.6 °C for *A. artemisiifolia* germination. These low *T*_min_ values may promote early germination in spring and thus prolong the growing season for this late-flowering annual. Furthermore, low *T*_min_ values should enable germination to occur even far north of the current distribution limit (Cunze et al. 2013) of the species.

### Differences in germination traits between populations from the native and invasive ranges and the influence of seed mass

*G*_max_ and T50 differed significantly between the native and the invasive range, with considerably higher germination speed and higher germination rates in the populations from the invasive range. With seedling establishment being a critical stage in plants’ life cycles (Donohue et al. 2010), especially individuals with fast germination and high germination rates may be able to compete with other species more effectively. High germination rates contribute significantly to invasion success (Mandak 2003; Radford and Cousens 2000).

Also for other species, differences in germination traits between native and introduced populations were found (reviewed in Donohue et al. 2010). In some cases, the introduced populations had a wider range of conditions under which germination was possible, or they germinated faster (Blair and Wolfe 2004; Cervera and Parra-Tabla 2009; Erfmeier and Bruelheide 2005), which we also observed for *A. artemisiifolia*. This finding can be explained with the EICA hypothesis: in invasive ranges other biotic or abiotic factors often support invasive species compared with factors in the native ranges (Hierro et al. 2005) or natural enemies or pathogens that are missing (Blossey and Noetzold 1995), which enables individuals to perform better and, e.g., invest more in the seeds. This again may be the result of advantageous germination properties of the seeds (e.g., increased germination rates). Accordingly, for *A. artemisiifolia*, seed mass was on average higher in populations from the invasive range and had an influence on *T*_max_, *G*_max_ and frost tolerance. We conclude that the increased seed mass of the European populations contributes to their higher germination rates, increased seedling frost tolerance and higher *T*_max_ values. However, the lower *T*_min_ values and higher germination speed found in the invasive range cannot be explained by the increased seed mass.

In populations from the invasive range, *T*_min_ was on average lower, and *T*_max_ was higher, which results in an increased *T*_range_ for germination. This finding is in contrast to our expectation of a narrower niche in the European populations due to the potential absence of some genotypes which did not make it to Europe. A wider germination niche may correspond to a wider ecological niche or range, but this is controversial in the literature (Donohue et al. 2010). One possible explanation for the increased temperature niche is that we have evidence for multiple introductions of *A. artemisiifolia* from various points of origin (Genton et al. 2005; Gladieux et al. 2011), which may cause increased genetic and phenotypic variability. Recent studies on the invasive *Bromus tectorum* indicate that range expansion in the invasive range might be facilitated by the introduction of specialized genotypes (Merrill et al. 2012).

Alternatively, an increased temperature niche may have evolved as an adaptation to changed selection pressures in the invasive ranges. It has been suggested that traits linked with germination may evolve quickly (Donohue et al. 2010), and successful invasive species are able to evolve in response to novel selection pressure and radiate into diverse habitats (Sakai et al. 2001). Fast evolution may be facilitated by increased genetic diversity and additive genetic variance resulting from admixture of populations from different sources and multiple introduction events (Ellstrand and Schierenbeck 2000; Lavergne and Molofsky 2007; Lee 2002; Prentis et al. 2008).

Overall, we estimate that the invasive *A. artemisiifolia* populations are exhibiting traits that may result in a higher fitness: faster germination, a broader germination temperature niche, and higher germination rates. Also Hodgins and Rieseberg (2011) demonstrated a higher fitness for many growth characteristics for European *A. artemisiifolia* populations compared to North American populations. These authors suggest an adaptation to more competitive environments in the invasive range.

### Relationship between germination temperature demands and environmental variables

For many life history traits, such as flowering phenology and reproductive biomass, latitudinal clines have been reported for *A. artemisiifolia* for native and invasive ranges (Chun et al. 2011; Hodgins and Rieseberg 2011; Leiblein-Wild and Tackenberg, under review). However, in contrast to our expectation, we hardly found any evidence for the hypothesis that germination traits of *A. artemisiifolia* are related to environmental variables of the respective point of origin, neither in the native nor in the invasive range. The missing significance may be partly attributed to the small number of populations used in the germination experiment. On the other hand, for frost tolerance, we found strong and significant correlations with environmental variables using a comparable sample size. Possibly, local microclimatic conditions differing from the mean climate in the area are more relevant for germination traits. Therefore, the use of mean climatic values for the statistical analysis may have resulted in non-significant effects on germination traits. For other (weedy) plant species, correlations between germination traits and temperature at the point of origin were found (De Frenne et al. 2009; Xia et al. 2011). In contrast, we have no evidence for local adaptation of germination parameters to the climate in *A. artemisiifolia*. However, besides the climatic environment, also maternal effects or other environmental variables at the points of origin (e.g., soil conditions, biotic factors etc.) may influence seed germination of the offspring, but such factors have not been investigated in our study.

On the other hand, the broad temperature niche for germination (mean = 30.5 °C) given for each single population, and relatively high germination rates even under less favorable temperatures, might compensate for a lack of adaptation to specific temperatures prevailing at the points of origin. Moreover, selection due to other environmental factors may be stronger: possibly for annual late-flowering species selection to seedling frost tolerance—for which we found a very strong correlation with climatic parameters—is more important than adaptations in germination temperatures, specifically in northern and temperate latitudes.

### Frost tolerance of seedlings

Generally, the mortality of seedlings exposed to frost was relatively high but comparable to those of different *Impatiens* species (Skálová et al. 2011). Frost tolerance of European populations was significantly higher, maybe due to the higher seed mass, which was positively related to seedling frost tolerance. This increased frost tolerance may be helpful for the north and eastwards range expansion since frost tolerance of seedlings is highly associated with the establishment phase of invasion (Skálová et al. 2011). An increased frost tolerance may enable early germinating individuals to survive late frost and to gain an advantage in fitness over late germinating individuals: early germination is accompanied by a prolongation of the growing period and may thus increase biomass accumulation. This may result in a higher pollen load and increased seed production, since these parameters are positively correlated with biomass in *A. artemisiifolia* (Fumanal et al. 2007). For example, *A. artemisiifolia* individuals that germinate in April achieve a final height of 170–180 cm and produced 3,000–4,000 achenes. For seeds germinating in August, the plant height is 10 cm with only 4.5 achenes produced (Dickerson and Sweet 1971; Kazinczi et al. 2008).

### Relationship between frost tolerance and environmental variables

Frost tolerance was correlated with the frost risk at the point of origin for North American populations. The extraordinarily high correlation coefficient (Spearman *ρ* = 0.73, *P* = 0.007) points to an important role of frost for the establishment of *A. artemisiifolia*. For many plant species frost resistance is correlated with local climatic conditions in the native range (Bannister and Polwart 2001), suggesting that populations from the native range have evolved frost tolerance as an evolutionary response to survive frost.

For European populations we found that frost tolerance differed between the populations, but was not correlated to the frost risk or temperature of the point of origin. Similarly, for *Buddleja davidii*, another invasive plant species, frost tolerance in the invasive range could not be related to geographic or climatic parameters at the point of origin of the populations (Ebeling et al. 2008). This obvious lack of local adaptation to low temperatures in the European populations probably reflects the fact that *A. artemisiifolia* is relatively new to Europe.

## Conclusion

We demonstrated that *A. artemisiifolia* germination takes place along a broad temperature gradient with high germination rates even under low temperatures, which may promote a high number of offspring nearly irrespective of the temperature at a given site. Different temperature demands for the germination of *A. artemisiifolia* between populations from invasive and native ranges indicate an expansion of the germination temperature niche in European populations which might be one reason for the species’ invasion success in Europe. Generally, seeds from European sites showed an increased fitness and robustness, i.e., higher germination rates, faster germination, and increased frost tolerance; all of these aspects favoring invasion success. The higher seed mass in the invasive range contributed to enhanced germination rates and higher *T*_max_ values, but did not contribute in the lower *T*_min_ values and higher germination speed of European ragweed populations. The lower *T*_min_ values associated with the increased frost tolerance of seedlings in the European populations may enable germination earlier in the year. This again might subsequently lead to higher biomass allocation due to a longer growing period (Donohue et al. 2010), which may be connected with an increased pollen and seed production (Fumanal et al. 2007). As a consequence, the existing medical problems with this highly invasive species might become more acute in Europe, and further invasion in Europe might be facilitated. As niche models predict an enlargement of suitable habitats for *A. artemisiifolia* under climate change conditions (Cunze et al. 2013), eradication measures should be intensified.

## Electronic supplementary material

Below is the link to the electronic supplementary material.

Supplementary material 1 (PDF 367 kb)
