# Supplementary material for: Germination and seedling frost tolerance differ between the native and invasive range in common ragweed
Source: Oecologia. 2013 Nov 7;174(3):739–50. doi: 10.1007/s00442-013-2813-6 (PMC3933736; doi:10.1007/s00442-013-2813-6)
Supplement: Supplementary file 1 — Supplementary material 1 (PDF 367 kb) [file 442_2013_2813_MOESM1_ESM.pdf]

Online Resource (Electronic Supplementary Material)

Article: Germination and seedling frost tolerance differ between the native and invasive range in common ragweed

Authors: Marion Carmen Leiblein-Wild, Rana Kaviani, Oliver Tackenberg

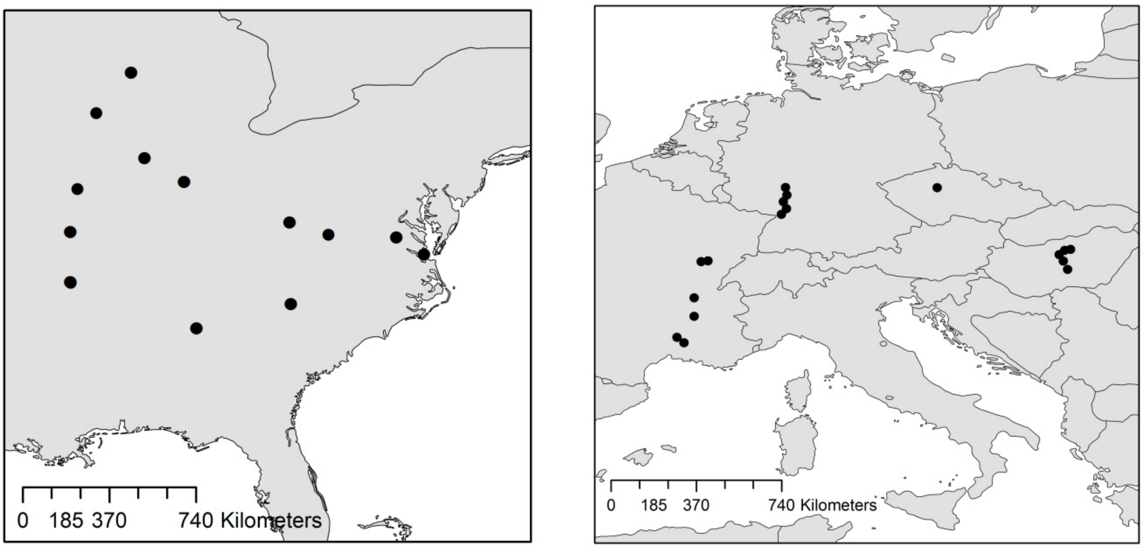

Fig. A1: Geographical points of origin of *A. artemisiifolia* populations from North America (left) and Europe (right) that were used in the germination experiment and the frost tolerance experiment.

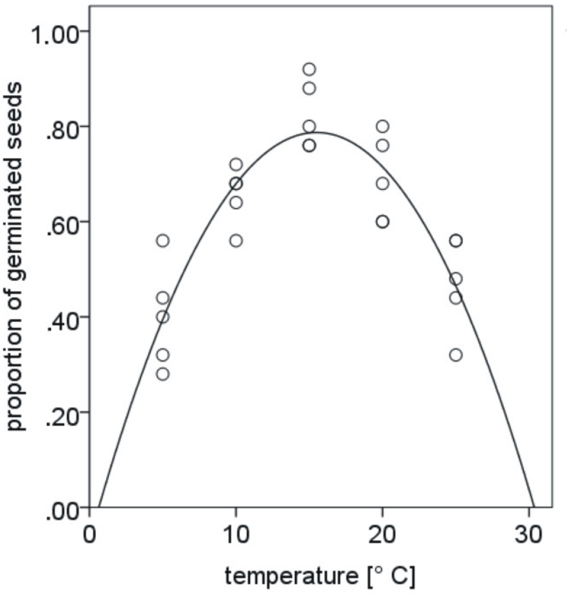

Fig. A2: Exemplary graph of a quadratic function for population E34 based on observations of germination rates in five temperature regimes and five replicates per regime after 60 days; circles: measured germination rates, line: graph of quadratic function ($y = -0.330 + 0.136x - 0.004x^2$, $R^2 = 0.77$, $P < 0.001$).

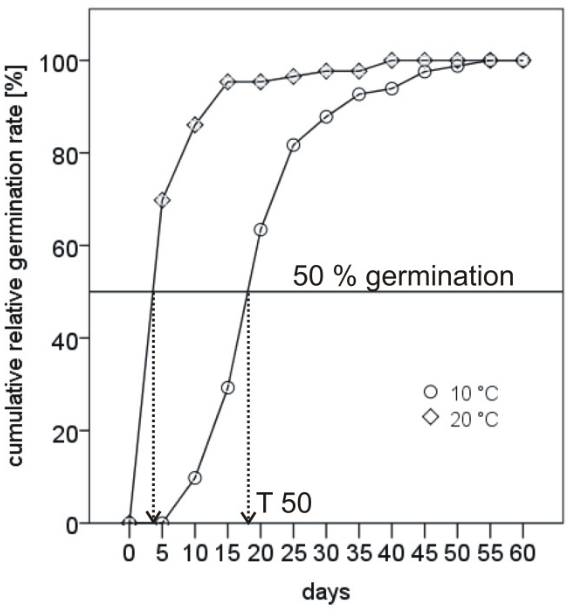

Fig. A3: Exemplary graph on calculation of T50 from cumulative germination rates of population E34 under two temperature regimes (10 °C and 20 °C). Final germination rate after 60 days in each temperature level (65.6 % at 10 °C, 68.8 % at 20 °C) was set to 100 %. T50 was calculated via linear interpolation.

Tab. A1: Values for R^2 and the predictors b0, b1 and b2 of the quadratic functions that describe the relationship between temperature and cumulative germination for all populations. Each function was based on observations of final germination rates in five temperature regimes and five replicates per regime after 60 days. For detailed information on each population, presented by its identification number (ID), see Tab. 1 in the manuscript.

PopID	b0	b1	b2	R^2	*P*
A9	-0.382	0.110	-0.003	0.838	< 0.001
A10	-0.550	0.143	-0.004	0.867	< 0.001
A16	-0.434	0.101	-0.003	0.759	< 0.001
A19	-0.378	0.084	-0.002	0.895	< 0.001
A21	-0.229	0.076	-0.002	0.839	< 0.001
A24	-0.342	0.087	-0.002	0.708	< 0.001
A28	-0.371	0.110	-0.004	0.809	< 0.001
A30	-0.282	0.089	-0.003	0.658	< 0.001
A36	-0.341	0.088	-0.003	0.638	< 0.001
A39	-0.304	0.095	-0.003	0.771	< 0.001
E2	-0.498	0.170	-0.006	0.810	< 0.001
E3	-0.143	0.124	-0.003	0.870	< 0.001
E6	-0.165	0.121	-0.003	0.872	< 0.001
E9	-0.330	0.136	-0.004	0.908	< 0.001
E12	-0.005	0.118	-0.003	0.884	< 0.001
E14	-0.150	0.116	-0.003	0.817	< 0.001
E17	-0.283	0.140	-0.004	0.734	< 0.001
E19	-0.502	0.149	-0.004	0.867	< 0.001
E20	-0.242	0.127	-0.003	0.851	< 0.001
E22	-0.442	0.147	-0.004	0.933	< 0.001
E23	-0.195	0.122	-0.003	0.914	< 0.001
E24	0.015	0.105	-0.003	0.828	< 0.001
E25	-0.346	0.152	-0.004	0.877	< 0.001
E27	-0.323	0.144	-0.004	0.796	< 0.001
E29	-0.355	0.129	-0.004	0.709	< 0.001
E30	-0.494	0.158	-0.004	0.946	< 0.001
E34	-0.069	0.110	-0.004	0.770	< 0.001
